# Glucocorticoid Resistance Syndrome as a Hidden Cause of Nonneoplastic Hypercortisolism

**DOI:** 10.1210/jendso/bvaf097

**Published:** 2025-05-20

**Authors:** Naoki Yamamoto, Nozomi Kido, Kenji Sugawara, Hironori Bando, Masaaki Yamamoto, Shin Urai, Yasutaka Tsujimoto, Yuka Ohmachi, Yuma Motomura, Yuka Oi-Yo, Yuriko Sasaki, Masaki Suzuki, Michiko Takahashi, Genzo Iguchi, Wataru Ogawa, Hidenori Fukuoka

**Affiliations:** Division of Diabetes and Endocrinology, Department of Internal Medicine, Kobe University Graduate School of Medicine, Kobe, Hyogo 650-0017, Japan; Division of Diabetes and Endocrinology, Department of Internal Medicine, Kobe University Graduate School of Medicine, Kobe, Hyogo 650-0017, Japan; Division of Diabetes and Endocrinology, Department of Internal Medicine, Kobe University Graduate School of Medicine, Kobe, Hyogo 650-0017, Japan; Division of Diabetes and Endocrinology, Department of Internal Medicine, Kobe University Hospital, Kobe, Hyogo 650-0017, Japan; Division of Diabetes and Endocrinology, Department of Internal Medicine, Kobe University Graduate School of Medicine, Kobe, Hyogo 650-0017, Japan; Division of Diabetes and Endocrinology, Department of Internal Medicine, Kobe University Hospital, Kobe, Hyogo 650-0017, Japan; Division of Diabetes and Endocrinology, Department of Internal Medicine, Kobe University Hospital, Kobe, Hyogo 650-0017, Japan; Division of Diabetes and Endocrinology, Department of Internal Medicine, Kobe University Graduate School of Medicine, Kobe, Hyogo 650-0017, Japan; Division of Diabetes and Endocrinology, Department of Internal Medicine, Kobe University Graduate School of Medicine, Kobe, Hyogo 650-0017, Japan; Division of Diabetes and Endocrinology, Department of Internal Medicine, Kobe University Graduate School of Medicine, Kobe, Hyogo 650-0017, Japan; Division of Diabetes and Endocrinology, Department of Internal Medicine, Kobe University Graduate School of Medicine, Kobe, Hyogo 650-0017, Japan; Division of Diabetes and Endocrinology, Department of Internal Medicine, Kobe University Graduate School of Medicine, Kobe, Hyogo 650-0017, Japan; Division of Diabetes and Endocrinology, Department of Internal Medicine, Kobe University Graduate School of Medicine, Kobe, Hyogo 650-0017, Japan; Division of Diabetes and Endocrinology, Department of Internal Medicine, Kobe University Hospital, Kobe, Hyogo 650-0017, Japan; Department of Nutrition, Kobe University Hospital, Kobe, Hyogo 650-0017, Japan; Division of Diabetes and Endocrinology, Department of Internal Medicine, Kobe University Graduate School of Medicine, Kobe, Hyogo 650-0017, Japan; Faculty of Clinical Nutrition and Dietetics, Department of Clinical Nutrition and Dietetics, Konan Women's University, Kobe, Hyogo 658-0001, Japan; Division of Diabetes and Endocrinology, Department of Internal Medicine, Kobe University Graduate School of Medicine, Kobe, Hyogo 650-0017, Japan; Division of Diabetes and Endocrinology, Department of Internal Medicine, Kobe University Hospital, Kobe, Hyogo 650-0017, Japan

**Keywords:** Cushing's syndrome, nonneoplastic hypercortisolism, glucocorticoid receptor, NR3C1, glucocorticoid resistance syndrome

## Abstract

Cases of hypercortisolemia without physical signs of Cushing's syndrome (CS), suggestive of nonneoplastic hypercortisolism (NNH), often remain partially unexplained. We present a unique case that was initially misdiagnosed as ACTH-dependent CS due to abnormal laboratory findings, despite the absence of Cushingoid features. Molecular and functional analyses ultimately led to a diagnosis of glucocorticoid resistance syndrome (GRS). A 54-year-old female patient underwent endocrinological evaluation for an adrenal incidentaloma associated with hypokalemia, which revealed hypercortisolemia. Subsequent endocrinological testing was consistent with ACTH-dependent CS; however, no Cushingoid features were observed on physical examination, suggesting NNH. As no apparent cause of NNH was identified, we hypothesized a functional disorder of the glucocorticoid receptor (GR) and performed a genetic analysis of *NR3C1*, which encodes GR. This revealed a novel germline heterozygous variant, p.L670P, located in the ligand-binding domain of the GR. Structural analyses revealed that Leu670 forms a hydrophobic core near the ligand-binding pocket. The p.L670P variant disrupted the secondary structure, suggesting a potential compromise in the structural stability of the ligand-binding site. In vitro experiments showed that this GR variant failed to suppress the transcriptional activity of the proopiomelanocortin promoter following dexamethasone administration. These findings confirmed that the patient had a loss-of-function variant in GR, leading to a diagnosis of GRS and ruling out ACTH-dependent CS. This case highlights that GRS may underline cases of NNH without a clear etiology, and genetic testing for GR can aid in its diagnosis.

Nonneoplastic hypercortisolism (NNH), also known as pseudo-Cushing's syndrome, should be differentiated from Cushing's syndrome (CS) in patients with hypercortisolism [1]. NNH is primarily caused by physiological hyperactivity of the hypothalamus-pituitary-adrenal (HPA) axis, typically triggered by conditions such as chronic alcoholism, obesity, eating disorders, chronic kidney disease, depression, and polycystic ovarian syndrome. Although extremely rare, glucocorticoid resistance syndrome (GRS), caused by genetic abnormalities in the glucocorticoid receptor (GR), can lead to NNH [2]. In patients with GRS who exhibit systemic resistance to glucocorticoids, impairment of the HPA axis negative feedback system results in hypercortisolism.

In contrast, endogenous CS results from neoplastic causes, such as cortisol-producing adrenal tumors and ACTH-producing tumors within or outside the pituitary [3]. Early diagnosis of CS is important because of its high mortality rate [4], and differentiating NNH from CS is crucial to avoid unnecessary treatment. However, this differentiation is often difficult, even for specialists, because of overlapping clinical and laboratory findings. Patients with NNH may exhibit some of the characteristic physical signs associated with CS; however, these signs may be absent in mild cases of CS [5]. Moreover, imaging studies are generally unhelpful for differentiation due to their insufficient sensitivity and specificity [3].

As valuable tests for differentiating CS from NNH, the desmopressin (DDAVP) test, the dexamethasone-corticotropin-releasing hormone (Dex-CRH) test, and midnight serum cortisol levels are known. In a recent meta-analysis, the DDAVP test was found to be the most specific test for distinguishing CS from NNH [6]. It has been reported that an increase in ACTH of 6 pg/mL or more after stimulation is considered consistent with CS, showing a sensitivity of 77% and specificity of 90%. Meanwhile, the Dex-CRH test is considered positive for CS when serum cortisol levels reach 1.4 µg/dL or higher at 15 minutes after CRH stimulation, demonstrating a sensitivity of 89% and specificity of 72%. Moreover, midnight serum cortisol levels greater than 7.5 μg/dL were also regarded as useful, with a sensitivity of 94% and specificity of 81% for diagnosing CS. However, there is still ongoing debate about their ideal cutoff values in various clinical contexts, and none have been established as a benchmark for distinguishing CS from NNH [1, 5, 6].

This report describes a case of NNH resulting from GRS, which is notable for the absence of adrenal hyperplasia and virilization, both of which are frequently associated with GRS. Furthermore, although the Dex-CRH test and late-night serum cortisol levels were consistent with CS, the identification of a loss-of-function variant in the GR ultimately led to the diagnosis of NNH due to GRS. Through this study, we demonstrate that GRS may be a hidden cause of NNH and underscore the utility of GR genetic analyses in distinguishing NNH from CS.

## Materials and Methods

### Ethics

This study adhered to the guidelines outlined in the Declaration of Helsinki and was approved by the Ethics Committee of Kobe University Graduate School of Medicine (approval nos. 1351 and 1646). The patient provided written informed consent for taking photographs, genetic analyses, and the publication of the results. A thorough investigation was performed to confirm this diagnosis.

### Hormone Assay

Serum cortisol and plasma ACTH levels were measured using a chemiluminescent immunoassay (Abbott Japan, Chiba, Japan; RRID: AB_2783639) and an electronic chemiluminescence immunoassay (Roche, Basel, Switzerland; RRID: AB_2783634) at the previous hospital. At our hospital, serum cortisol and plasma ACTH levels were measured using an enzyme immunoassay (TOSOH, Tokyo, Japan; RRID: AB_3076600) and an electronic chemiluminescence immunoassay (TOSOH, RRID: AB_2783633), respectively. Urinary-free cortisol levels were measured using an enzyme immunoassay (IRMA; TFB, Tokyo, Japan; RRID: AB_2894408).

### Evaluation of Hypercortisolemia

The low-dose dexamethasone suppression test (LDDST) involved the oral administration of 1 mg dexamethasone (Dex) at 23:00. The following morning, blood samples were collected 30 minutes after the patient rested in the supine position. Serum cortisol levels ≥1.8 μg/dL were used as the cutoff value for suspicion of CS [7]. In the DDAVP test, a single IV injection of DDAVP (4 μg) was administered following the guidelines of the Japan Endocrine Society [8]. Blood samples were collected before and at 30, 60, 90, and 120 minutes after DDAVP administration, and plasma ACTH levels were measured. A response was deemed significant if ACTH levels increased by >1.5 times the basal levels. For the Dex-CRH test, 0.5 mg Dex was orally administered every 6 hours for 48 hours, followed by an IV injection of 100 μg human CRH (Tanabe, Osaka, Japan), as previously described [9]. A serum cortisol concentration of >1.4 μg/dL, measured 15 minutes after CRH administration, was considered a positive response.

### DNA Sequencing and Analysis

Genomic DNA was extracted from whole blood using a Gentra Puregene Blood Kit (QIAGEN, Hilden, Germany) following the manufacturer's protocol. The purity and quantity of extracted DNA were assessed using a NanoDrop spectrophotometer (Thermo Fisher Scientific, Waltham, MA, USA). The *NR3C1* coding region was amplified from the extracted genomic DNA via polymerase chain reaction (PCR) using primers previously described in the literature [10]. The resulting PCR products were analyzed by Sanger sequencing using the aforementioned forward and reverse primers. The *NR3C1* variant identified in this study was submitted to ClinVar and is available under accession number SCV005908114.

### Cell Culture

Human embryonic kidney (HEK293T) cells were obtained from the American Type Culture Collection (Manassas, VA, USA; RRID: CVCL_0063). The cells were cultured in Dulbecco's Modified Eagle's Medium (Gibco, Grand Island, NY, USA) supplemented with 10% fetal bovine serum (Gibco) at 37 °C in a 5% CO_2_ environment.

### Plasmid Construction

RNA was first extracted from healthy human blood using NucleoSpin RNA Blood kit (MACHEREY-NAGEL, Düren, Germany), followed by reverse transcription with the ReverTra Ace qPCR RT Master Mix with gDNA Remover (TOYOBO). Using the resulting cDNA as a template, reverse transcription PCR targeting human GRα was performed with the following primers: forward: 5′-CACCATGGACTCCAAAGAATCAT-3′, reverse: 5′-CTTTTGATGAAACAGAAGTTTTTTG-3′. The PCR product was cloned into the pcDNA3.1-D/V5-His-TOPO vector to create a plasmid expressing wild-type human GRα (NM_000176.3) (hGRα WT). Plasmids expressing human GRα with the p.L670P (c.2009 T > C) (hGRα L670P) and p.L672P (c.2015 T > C) (hGRα L672P) variants were generated using the PrimeSTAR Mutagenesis Basal Kit (TaKaRa, Kusatsu, Japan) with hGRα WT as a template. The following primers were used: hGRα L670P forward: 5′-ACCTTACCGCTTCTCTCTTCAGT-3′, hGRα L670P reverse: 5′-GAGAAGCGGTAAGGTTTTCATACAGA-3′; hGRα L672P forward: 5′-GGAATGACGAAGGGAGAA-3′, hGRα L672P reverse: 5′-AAGAGGGAAGCAGTAAGG-3′. The rat *proopiomelanocortin* (*Pomc*) promoter (−480/+63 region) was kindly provided by Prof. Shlomo Melmed (Cedars-Sinai Medical Center, Los Angeles, CA, USA) and cloned into the pGL4.10 [luc2] vector (cat. no. E6651, Promega), as described previously [11]. This construct is referred to as *Pomc-Luc* hereafter.

### Western Blotting

To confirm the expression of hGRα in HEK293T cells, proteins were extracted from cell lysates at 4 °C 48 hours after transfection with the corresponding plasmids. Then, 8 μg of the protein extracts were subjected to electrophoresis, and immunoblotting was performed using a mouse monoclonal antibody targeting GRα (1:100, Santa Cruz, CA, cat. no. sc-12763, RRID: AB_627675). Horseradish peroxidase-conjugated anti-mouse IgG (1:5000, Thermo Fisher Scientific, cat. no. 62-6520, RRID: AB_2533947) was used as the secondary antibody. Horseradish peroxidase-conjugated anti-b-actin antibody (1:100,000, Abcam, cat. no. ab49900, RRID: AB_867494) served as the internal control.

### Structural Analysis

The coordinates for the GR ligand-binding domain (LBD) complexed with Dex were obtained from the Protein Data Bank (PDB; 1M2Z [12]). The 3-dimensional structures of the wild-type and p.L670P variant LBDs were predicted using the AlphaFold 3 server [13]. Following structure prediction, molecular dynamics simulations were performed using GROMACS [14], applying the OPLS-AA force field and the TIP3P water model. Energy minimization and system equilibration were carried out before running simulations under isothermal–isobaric conditions for 5 ns. All structural figures were generated using the PyMOL Molecular Graphics System (Version 3.1, Schrödinger, LLC). PyMOL was also used to calculate the backbone root mean square deviation values and to define and visualize secondary structure elements.

### Luciferase Reporter Assay

HEK293T cells were seeded at a density of 5.0 × 10^4^ cells per well in 24-well plates and transfected with expression plasmids encoding for hGRα WT, hGRα L670P, or hGRα L672P (0.8 mg/well, respectively), along with *Pomc*-*luc* (0.8 μg/well) and pNL1.1.PGK (1 ng/well), which contains the NanoLuc reporter. Transfection was carried out using Lipofectamine 2000 (Invitrogen, Waltham, MA, USA). The empty vector, pGL4.10 [luc2], served as a negative control. After 24 hours of transfection, the cells were treated with either vehicle (dimethyl sulfoxide) or Dex at concentrations of 10 nM or 100 nM for 24 hours. Cells were then harvested, and luciferase activity was measured using the Nano-Glo Dual-Luciferase Assay System® (Promega, Madison, WI, USA), following a previously reported method [15]. Luciferase activity was measured using an EnSpire™ Multimode Plate Reader (PerkinElmer, Waltham, MA, USA) and normalized to the internal NanoLuc activity. All experiments were performed in triplicate and repeated twice for reproducibility.

### Statistical Analysis

Results are expressed as the mean and SD. All statistical analyses were performed using SPSS version 29.0 (IBM Corporation, Armonk, NY, USA). Differences between groups in the luciferase reporter assay were evaluated using 1-way ANOVA followed by the post hoc Tukey–Kramer test. Statistical significance was defined as *P* < .05.

## Results

### Case Presentation

**Figure 1.**
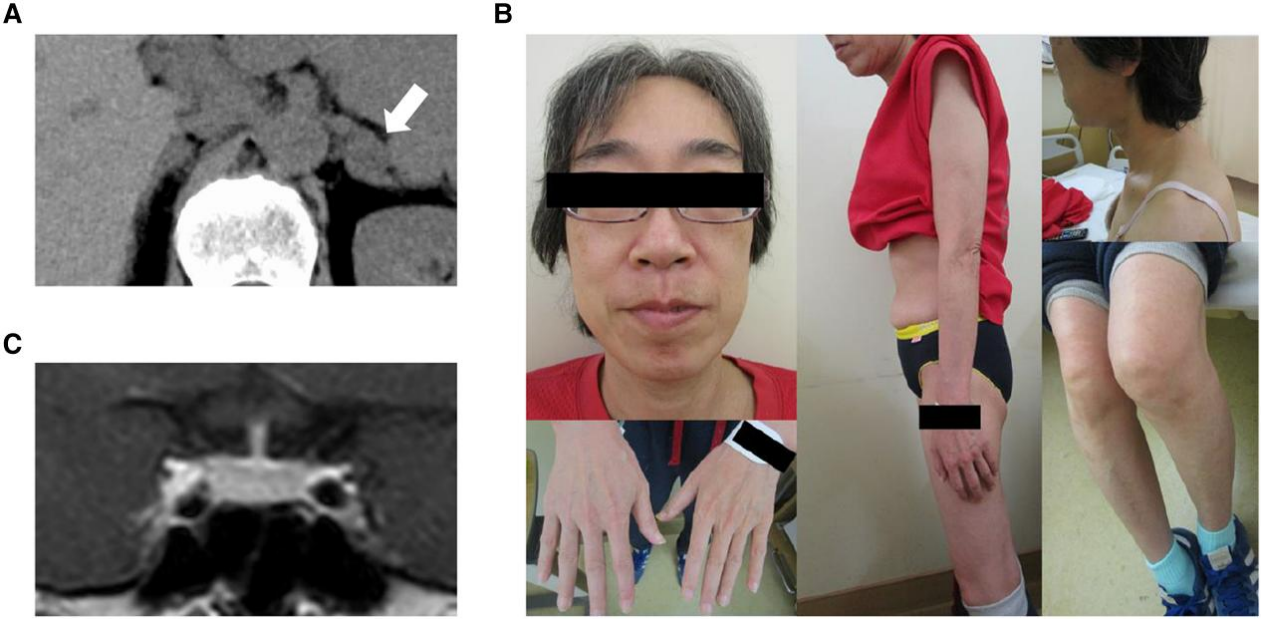
Clinical findings on patient imaging and physical examination. (A) Findings of abdominal CT scan. The white arrow indicates the left adrenal tumor, which is 20 mm in size and has a CT value of 10 HU. (B) Photo of the patient. No signs suggestive of Cushingoid symptoms, including moon face, central obesity, purple striae, skin atrophy, proximal myopathy, peripheral edema, or hirsutism, are observed. (C) MRI of the pituitary gland with a contrast reagent. Abbreviations: CT, computed tomography; HU, Hounsfield units; MRI, magnetic resonance imaging.

A 47-year-old woman developed diabetes mellitus, which was diagnosed as type 1 diabetes mellitus (T1DM) based on a markedly reduced level of endogenous insulin secretion despite being negative for anti-glutamic acid decarboxylase antibody. At the age of 54, she was hospitalized at the previous hospital for diabetic ketoacidosis. During treatment, she developed transient hypokalemia (2.8 mmol/L), and noncontrast computed tomography (CT) revealed a left adrenal incidentaloma measuring 20 mm with low density (10 Hounsfield units), without evident atrophy of the contralateral adrenal gland (Fig. 1A). Although the adrenal tumor was considered a benign adrenocortical adenoma based on its low CT attenuation and homogeneous appearance, these findings prompted further endocrinological evaluation. As a result, her serum cortisol levels were elevated (26.5 µg/dL), with unsuppressed plasma ACTH levels (27.0 pg/mL), raising suspicion of ACTH-dependent CS. The patient was referred to our hospital for further investigation. Her growth and developmental histories were unremarkable, and she reported no specific menstrual abnormalities until menopause at 53 years of age. Her medical history included hypertension since her late 40s and an atherosclerotic stroke at 53 years of age. She had a family history of hypertension in her father. She was being treated with insulin, calcium channel blockers, angiotensin II receptor blockers, and statins; however, none of these are known to affect CYP3A4 activity or cortisol-binding globulin levels. She did not consume alcohol and had no apparent history of depression. The patient was lean, with a height of 159.0 cm and a body mass index of 18.7 kg/m^2^, without significant recent changes in body weight. Importantly, she exhibited no physical signs of CS, including moon face, central obesity, purple striae, skin atrophy, proximal myopathy, peripheral edema, or virilization (Fig. 1B). Similarly, laboratory examinations revealed no hypercortisolemia-related abnormalities, such as neutrophilia or electrolyte imbalances, except for a high hemoglobin A1c level (8.9%). Contrast-enhanced magnetic resonance imaging did not reveal any obvious pituitary mass (Fig. 1C). However, urinary collection test showed high urinary free cortisol levels of 98.1 μg/day (reference range: 5.5-66.7 μg/day), suggestive of excess cortisol secretion. Moreover, the patient's morning serum cortisol levels after the LDDST were not suppressed (8.3 μg/dL). Late-night plasma ACTH and serum cortisol levels were 22.0 pg/mL and 8.7 μg/dL, respectively, indicating autonomous cortisol secretion in an ACTH-dependent manner. Despite the presence of hypercortisolemia, the absence of physical findings suggestive of CS raised the possibility of NNH. To differentiate between NNH and CS, the DDAVP and Dex-CRH tests were performed. In the DDAVP test, the patient's plasma ACTH levels showed minimal change (baseline: 32.9 pg/mL; peak: 39.5 pg/mL at 120 minutes), a result consistent with NNH. In the Dex-CRH test, the serum cortisol level 15 minutes after CRH administration was 1.6 μg/dL, which exceeded the 1.4 μg/dL threshold for suspicion of CS [1]. However, the inadequate suppression of plasma ACTH (13.7 pg/mL) and serum cortisol (1.2 μg/dL) suggested NNH associated with resistance to Dex, rather than CS. Despite these findings, the patient exhibited no common causes of NNH, such as chronic alcoholism, obesity, depression, or chronic kidney disease. In addition, hypercortisolemia without Cushingoid features and unsuppressed serum cortisol levels in both the Dex suppression and Dex-CRH tests indicated resistance to glucocorticoid effects. Consequently, the possibility of GRS was considered as a rare cause of NNH, prompting genetic analyses of *NR3C1,* the gene encoding GR.

### Genetic Analysis

**Figure 2.**
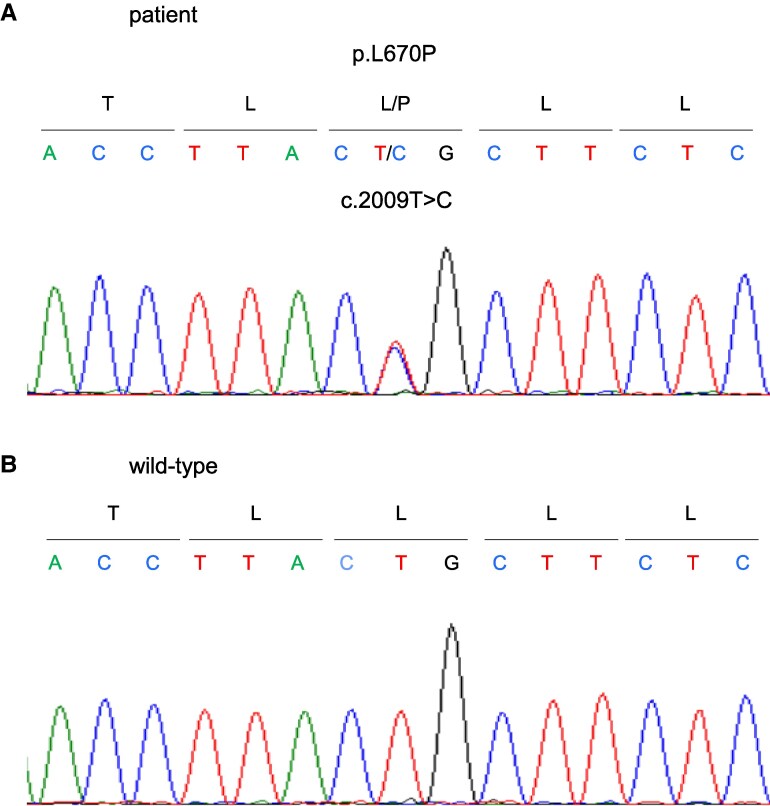
Results of *NR3C1* gene analysis by Sanger sequencing. The c.2009 T > C (p.L670P) variant is detected in the patient's whole blood. (A) Patient and (B) wild-type (healthy subjects).

Sanger sequencing revealed a heterozygous variant, c.2009 T > C, in exon 7 of *NR3C1* (NM_000176.3) (Fig. 2A and 2B). This novel variant results in the substitution of the 670th leucine with proline (p.L670P), located within the LBD of the GR. Genetic analyses of family members were not performed due to lack of consent. To assess the potential functional impact of the variant on GR, 4 in silico predictive models were used: Polymorphism Phenotyping 2 [16], Sorting Intolerant From Tolerant [17], Mutation Taster [18], and GVGD Alignment [19]. All models predicted that this variant would impair protein function. According to the consensus recommendation of the American College of Medical Genetics and Genomics [20], the variant was classified as “likely pathogenic” (class III).

### Structural Analysis

**Figure 3.**
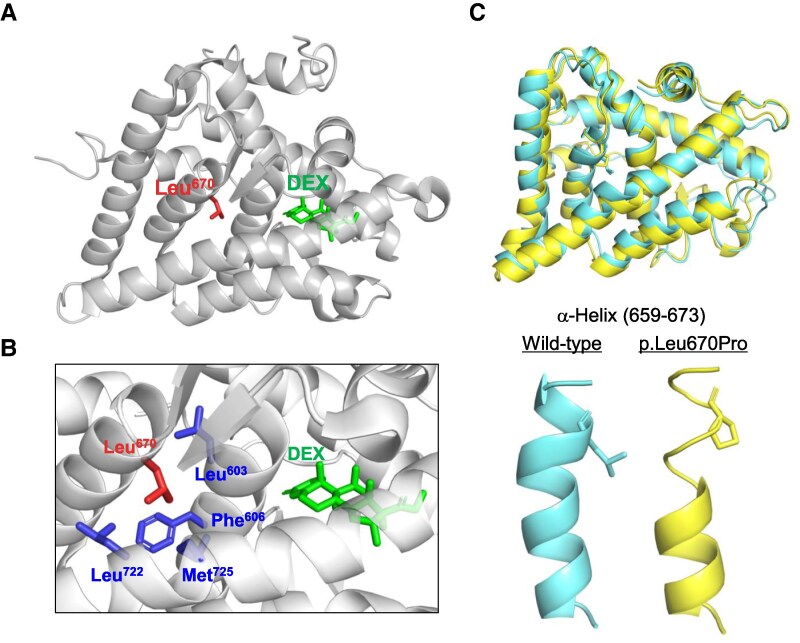
Structural presentation of Leu670 in the LBD and the impact of the p.L670P variant. (A) Overall structure of the LBD bound to Dex, based on the crystal structure of the LBD–Dex complex (PDB: 1M2Z). Leu670 and Dex are highlighted and indicated by labels. (B) Close-up view of the Dex-binding pocket, showing that Leu670 forms a hydrophobic core with Leu603, Phe606, Leu722, and Met725, all of which are labeled accordingly. (C) Predicted structures of the wild-type and p.L670P variant. Both structures were first generated by AlphaFold 3, followed by 5 ns of molecular dynamics simulations using GROMACS. The α-helical region around residues 659 to 673 is shown in the lower panels. Abbreviations: Dex, dexamethasone; LBD, ligand binding domain.

The crystal structure of the LBD of the GR-Dex complex (PDB: 1M2Z [12]) showed that Leu670 was located near the Dex-binding pocket (Fig. 3A). Leu670 forms a hydrophobic core with Leu603, Phe606, Leu722, and Met725 (Fig. 3B), residues that are highly conserved across species. This conservation highlights the critical role of Leu670 in maintaining the 3-dimensional structure of the ligand-binding pocket.

To assess the impact of the p.L670P variant on the 3-dimensional structure, we utilized the AlphaFold 3 server [13] to predict the structures of both the wild-type and p.L670P variant, followed by molecular dynamics simulations. Our findings revealed that the overall structure of LDB exhibited no major differences between the wild-type and variant, with a root mean square deviation of 1.43 Å (Fig. 3C, upper). However, the p.L670P variant caused a loss of the α-helical structure in the region spanning residues 659 to 673 after 5 ns of simulation (Fig. 3C, lower). In contrast, the wild-type structure remained stable throughout the simulation. Taken together, these findings suggest that the p.L670P variant significantly disrupts the secondary structure and may compromise the stability of the steroid-binding pocket in the LBD.

### Functional Analysis of GR

**Figure 4.**
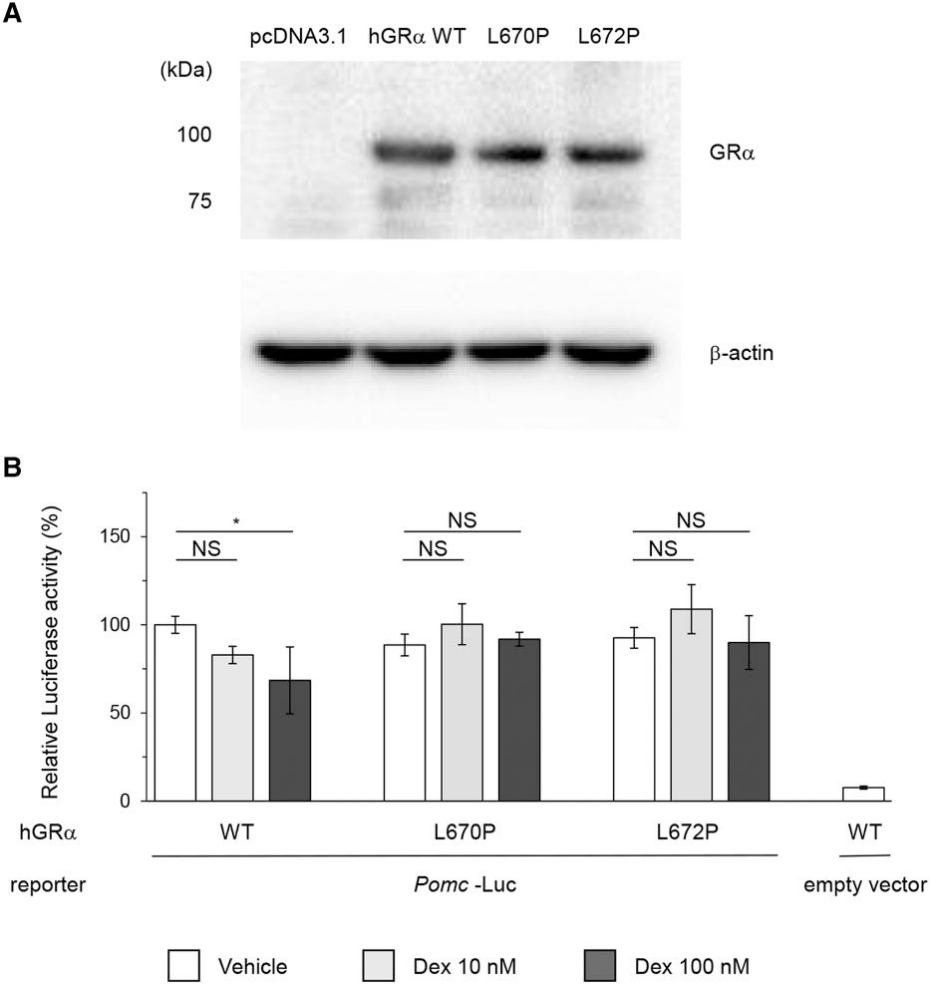
Functional analysis of the hGRα p.L670P variant. (A) Expression of the hGRα protein from each plasmid is confirmed. HEK293T cells are transfected with 0.8 μg plasmids encoding for hGRα WT, hGRα L670P, or hGRα L672P. The empty vector, pcDNA3.1, is used as a negative control. Transfected cells are lysed, and protein extracts are analyzed by SDS-PAGE. hGRα proteins are detected by Western blotting using a mouse monoclonal antibody. (B) Functional analysis of the hGRα variant. HEK293T cells are transfected with expression plasmids encoding for hGRα WT, hGRα L670P, or hGRα L672P, along with *Pomc*-*luc.* The empty vector, pGL4.10 [luc2], serves as a negative control. Transfected cells are subsequently treated with vehicle, Dex 10 nM, or Dex 100 nM for 24 hours, and *Pomc*-promoter activity is analyzed using a luciferase reporter assay. Normalized *Pomc-* promoter activity (luciferase signal corrected by NanoLuc internal control) in cells expressing hGRα wild-type and treated with vehicle was defined as 100%, corresponding to a mean relative luciferase value of 17.22 ± 1.96. Bars indicate the mean values of relative luciferase activity, and error bars indicate SDs. Statistical significance is calculated using one-way ANOVA with post hoc Tukey–Kramer test. *P < .05. Abbreviations: Dex, dexamethasone; hGRα, human glucocorticoid receptor α; NS, not significant.

We hypothesized that the novel GRα p.L670P variant impairs GR function, thereby disrupting the negative feedback regulation of adrenal cortisol synthesis and pituitary ACTH production. To investigate this, we examined whether the promoter activity of *Pomc*—encoding the ACTH precursor—was differently suppressed by Dex administration in cells expressing the wild-type GR vs the variant. We constructed vectors expressing hGRα WT, hGRα L670P, or hGRα L672P; the latter was a previously reported functionally impaired variant [21]. The expression of hGRα was confirmed by Western blotting (Fig. 4A). Following transfection of HEK293T cells with each vector expressing hGRα, along with *Pomc*-*luc*, the cells were treated with either vehicle or Dex, and *Pomc* promoter activity was assessed. In cells expressing hGRα WT, *Pomc* promoter activity was significantly suppressed after treatment with 100 nM Dex compared to vehicle (*P* = .01). In contrast, cells expressing hGRα L670P showed a complete disruption of Dex-induced suppression of *Pomc* promoter activity at concentrations of 10 and 100 nM (*P* = .08, *P* = .88, respectively), consistent with the results observed in cells expressing hGRα L672P (Fig. 4B). These findings demonstrate that the *NR3C1* variant p.L670P leads to a loss of GR function. This is consistent with the observed phenotype of elevated ACTH and cortisol levels and the impaired Dex-induced suppression noted in this case.

## Discussion

Based on the results of the *NR3C1* genetic and functional analyses, this patient was considered a suspected case of NNH and was ultimately diagnosed with GRS. Diagnosing asymptomatic hypercortisolism involves several endocrinological and imaging tests, culminating in the analysis of the *NR3C1* gene to identify underlying abnormalities. We believe that this straightforward genetic test offers valuable insights into the differential diagnosis of NNH and CS, which could be useful in some cases.

Among the dynamic tests used to distinguish NNH from CS [6], the absence of a reactive increase in ACTH during the DDAVP test was consistent with the diagnosis of NNH rather than CS. However, in the Dex-CRH test, serum cortisol levels after CRH administration exceeded the threshold, suggesting CS. This may be attributed to the failure of Dex to adequately suppress serum cortisol levels before the CRH infusion, likely due to glucocorticoid resistance. These findings suggest that the Dex-CRH test may have limited utility in differentiating CS from NNH associated with the GRS. In addition, the diagnostic utility of the Dex-CRH test has been debated in recent years. As noted by Findling and Raff, some experts consider this test to be of limited current value, as it may yield abnormal results in both CS and NNH, thereby limiting its specificity [2]. The present case suggests that false-positive results may occur even in GRS, further questioning the reliability of this test. Furthermore, while the DDAVP test has emerged as a useful adjunct in distinguishing CS from NNH, caution is warranted, as a small proportion of patients with ectopic ACTH-producing tumors may also exhibit a robust ACTH response [22]. These considerations highlight the interpretive challenges of dynamic testing for diagnosing CS. In the present case, however, the identification of the loss-of-function *NR3C1* variant allowed for the diagnosis of GRS and ruled out the possibility of CS.

Importantly, this case exhibited neither the physical signs of CS nor the common clinical presentations associated with GRS. Since 1976, more than 30 variants of *NR3C1* have been reported to cause GRS, with these variants widely distributed across all GR domains, with no known hot spots [23]. Although most GRS cases are diagnosed in adulthood, the clinical phenotype is highly variable, likely due to differences in the severity of glucocorticoid resistance in each case. Generalized resistance to glucocorticoids impairs the negative feedback system of the HPA axis, leading to chronic ACTH excess, which in turn stimulates adrenal steroidogenesis. This causes excessive secretion of mineralocorticoids and androgens. Common clinical signs of GRS include adrenal hyperplasia and hirsutism in women, which have been reported in 81.3% and 76.5% of patients, respectively [24]. It should be noted that both these signs can also be observed in CS [25]. Additionally, variants of *NR3C1* have been detected in 5% of patients with bilateral adrenal hyperplasia associated with hypercortisolemia and hypertension [26], emphasizing the importance of adrenal hyperplasia in the diagnosis of GRS.

However, adrenal hyperplasia and hirsutism were not observed in the present case. The relatively mild resistance to glucocorticoids and modest excess ACTH in this case might explain the phenotype. This seems to contradict the fact that the GR, with the variant in this case, showed complete resistance to Dex in vitro. Interestingly, however, the clinical severity of glucocorticoid resistance is not considered to be associated with the in vitro functionality of the variant GR or genetic factors, such as variant localization or whether the variant is homozygous or heterozygous. This case suggests that GRS may be concealed in patients with NNH, even in the absence of typical clinical signs of CS.

The patient had hypertension, which might be a complication of the GRS. If refractory hypertension, hypokalemia, or virilization develops, glucocorticoids without mineralocorticoid action, such as Dex, may be a treatment option. This medication may help inhibit the excessive secretion of ACTH, which in turn reduces the overproduction of adrenal steroids that exhibit mineralocorticoid and androgenic effects [27].

Most *NR3C1* variants are familial, although sporadic variants have also been reported [24]. In this case, genetic testing of the patient's family members was not performed after discussion with the patient. Although there was no apparent family history suggestive of GRS, it remains unclear whether GRS exists in the family, as clinical symptoms were absent in this case.

Here, we show that the novel *NR3C1* variant p.L670P observed in this case did not suppress *Pomc* promoter activity upon Dex administration, indicating a loss-of-function of GR. Furthermore, structural analysis predicted that Leu670 is located near the Dex-binding site of GR and that the mutation to proline disrupts the stability of the steroid-binding pocket of the LBD. Therefore, it is suggested that the L670P variant causes a loss-of-function of GR by impairing ligand binding to the LBD, which is the likely cause of GRS in this case.

Interestingly, our patient had a coexistence of T1DM, which raises the question of a possible link between GRS and autoimmune disease. Although the association between GRS and T1DM remains unclear, there have been recent reports of patients with GRS who also developed T1DM [28]. Moreover, certain *NR3C1* polymorphisms associated with altered GR sensitivity or resistance have been reported to potentially contribute to the risk of developing specific autoimmune diseases [29-32]. Although a direct causal relationship cannot be established, systemic glucocorticoid resistance in this case may have increased the susceptibility to autoimmunity and potentially contributed to the development of T1DM. Further accumulation and investigation of similar cases will be necessary to clarify the potential link between GRS and autoimmune diseases, including T1DM.

Moreover, in the present case, although typical adrenocortical hyperplasia commonly observed in GRS was not present, a unilateral adrenal incidentaloma was identified. Several previous reports have described the presence of adrenal incidentalomas in patients with GRS [21, 33], including a recent case in which an adrenal adenoma with mild autonomous cortisol secretion was associated with suppressed ACTH levels prior to adrenalectomy [28]. Because of the underlying glucocorticoid resistance, the interpretation of the LDDST is inherently limited, and autonomous cortisol secretion from the adrenal lesion in our patient cannot be entirely excluded; however, there was no evidence of suppressed ACTH secretion or contralateral adrenal atrophy, and thus no clear indication of functional cortisol excess was observed. Although the adrenal tumor in this case was considered a benign adrenocortical adenoma based on its low CT attenuation and homogeneous appearance, and routine imaging follow-up may not be mandatory [34], adrenalectomy should be considered if evidence of increased autonomous cortisol secretion emerges.

In conclusion, a patient with ACTH-dependent CS-like endocrinological findings but without any clinical symptoms of CS was diagnosed with GRS. GRS may also be present in patients with suspected NNH. In such cases, early genetic analysis can help rule out CS, avoiding invasive testing and unnecessary therapeutic interventions. In individuals with hypercortisolemia who lack physical symptoms of CS and have no common etiologies of NNH, such as chronic alcoholism, depression, or obesity, it may be prudent to consider GRS as a rare potential cause. Further studies are needed to clarify the frequency of hidden GRS in patients presenting with NNH.

## Data Availability

Some or all datasets generated during and/or analyzed during the current study are not publicly available but are available from the corresponding author on reasonable request.

## Abbreviations

CS: Cushing's syndrome
CT: computed tomography
DDAVP: desmopressin
Dex: dexamethasone
GR: glucocorticoid receptor
GRS: glucocorticoid resistance syndrome
hGRα: human glucocorticoid receptor α
HPA: hypothalamus-pituitary-adrenal
LBD: ligand-binding domain
LDDST: low-dose dexamethasone suppression test
NNH: nonneoplastic hypercortisolism
PCR: polymerase chain reaction
PDB: Protein Data Bank
T1DM: type 1 diabetes mellitus
